# Comprehensive assessment of pain characteristics, quality of life, and pain management in cancer patients: a multi-center cross-sectional study

**DOI:** 10.1007/s11136-024-03725-w

**Published:** 2024-08-06

**Authors:** Sunil Shrestha, Simit Sapkota, Siew Li Teoh, Bhuvan KC, Vibhu Paudyal, Shaun Wen Huey Lee, Siew Hua Gan

**Affiliations:** 1 School of Pharmacy, Monash University Malaysia, Jalan Lagoon Selatan, Bandar Sunway, Selangor 47500 Malaysia; 2 Department of Clinical Oncology, Kathmandu Cancer Center, Tathali, Bhaktapur, Bagmati Province Nepal; 3 Department of Clinical Oncology, Civil Service Hospital, Minbhawan, Kathmandu, Bagmati Province Nepal; 4 School of Clinical Sciences, Queensland University of Technology, Brisbane, Australia; 5 College of Public Health, Medical, and Veterinary Sciences, James Cook University, Townsville, QLD Australia; 6 School of Pharmacy, College of Medical and Dental 21 Sciences, Sir Robert Aitken Institute for Medical Research, University of Birmingham Edgbaston, Birmingham, B15 2TT UK; 7 Florence Nightingale Faculty of Nursing, Midwifery and Palliative Care, King’s College London, London, UK; 8 Asian Centre for Evidence Synthesis in Population, Implementation and Clinical Outcomes (PICO), Health and Well Being Cluster, Monash University Malaysia, Bandar Sunway, Selangor Malaysia; 9 Global Asia in the 21st Century (GA21) Platform, Monash University Malaysia, Bandar Sunway, Selangor Malaysia

**Keywords:** Cancer pain, Quality of life, Pain intensity, Pain severity, Medication adherence

## Abstract

**Introduction:**

Pain is the most common complaint among cancer patients, significantly impairing their health-related quality of life (HRQOL). There is limited evidence on the characteristics of pain among cancer patients in Nepal with low-resource settings.

**Objectives:**

The primary objective of this study was to evaluate the clinical characteristics of pain, factors influencing pain intensity, and the association of pain severity with quality of life (QoL) among cancer patients. Secondary objectives included investigating perceived barriers to pain management and medication adherence among these patients.

**Methods:**

This multi-center, cross-sectional study enrolled adult patients (over 18 years old) with reported cancer diagnoses experiencing pain. Socio-demographic characteristics (e.g., age, gender, educational status), clinical characteristics (e.g. cancer diagnosis, staging), and pain characteristics (e.g., duration, type, location, medicines used for pain management, etc.) were recorded. Outcomes were assessed using the Numeric rating scale (NRS), Pain management Index, European Organization for the Research and Treatment of Cancer Quality of Life Questionnaire, Barriers Questionnaire II, Medication Adherence Rating Scale, and Hospital Anxiety and Depression Scale.

**Results:**

Four hundred and eight patients participated in the study. The mean ± SD age was 54.87 ± 15.65, with 226 patients (55.4%) being female. The most common cancer diagnoses were cervical (17.6%), lung (11.8%), and colon/rectum (12.0%) cancers. The most common pain locations were the head and neck (27.0%); a majority (55.6%) reported pain duration of more than 3 months. Nociceptive pain was reported by 42.4% of patients; the mean ± SD of NRS was 4.31 ± 2.69, with 32.4% of patients experiencing moderate pain. Patients with mixed pain type (B = 1.458, *p* < 0.001) or pain in multiple sites (B = 1.175, *p* < 0.001), lower Karnofsky Performance Status (KPS) (B = -1.308, *p* < 0.001), and specific cancer diagnoses such as prostate (B = -2.045, *p* = 0.002), pancreatic (B = 1.852, *p* = 0.004), oesophageal (B = 1.674, *p* = 0.012), and ovarian cancer (B = 1.967, *p* = 0.047), experienced varying degrees of increased NRS score. The combined chemotherapy and radiotherapy treatment modality was associated with a lower NRS score (B = -0.583, *p* = 0.017). A significant inverse relationship was observed between pain severity and global health status/QoL (B = -37.36, *p* < 0.001. Key barriers to pain management included moderate perceptions of physiological effects, communication issues between doctors and patients, and concerns about the harmful effects of pain medicine. The prevalence of non-adherence to pain medications was 13.97%.

**Conclusion:**

In conclusion, this study highlights the multi-faceted nature of pain management and QoL for cancer patients in Nepal with low-resource settings. These findings underscore the multifactorial nature of pain perception in cancer patients, with mixed pain types, pain in multiple sites, lower KPS, and specific cancer diagnoses, all contributing significantly to pain severity. Additionally, pain severity was associated with declining QoL. These findings contribute valuable insights into the complex aspects of cancer pain and its broader implications for the well-being of patients, offering a foundation for targeted interventions and improved pain management strategies in the context of cancer care in low-resource settings.

**Supplementary Information:**

The online version contains supplementary material available at 10.1007/s11136-024-03725-w.

## Introduction

Among non-communicable diseases (NCDs), cancer ranks as a leading cause of death worldwide, posing a substantial barrier to improvements in life expectancy [1]. According to GLOBOCAN 2022, published by the International Agency for Research on Cancer (IACR), there were an estimated 20 million new cancer cases and approximately 10.0 million cancer deaths in 2022 [2]. Cancer pain, a widespread global issue [3], encompasses nociceptive (arising from tissue damage or inflammation) or neuropathic sources [4–6]. Such pain poses a significant challenge for patients, extending beyond the initial diagnosis to include managing physical and psychological treatment effects [3, 7–9]. The failure to achieve adequate pain control is demonstrably associated with a cluster of adverse consequences, encompassing significant patient distress, impaired physical and emotional functioning, and a diminished quality of life (QoL) for both the individual and their families [9]. The World Health Organization (WHO) defines QoL as “*an individual’s perception of their position in life in the context of the culture and value systems in which they live and in relation to their goals, expectations, standards, and concerns”* [10]. Cancer pain has an adverse impact on several well-being categories, including emotional state [11, 12], social interactions [13], physical symptoms [14], and even spirituality [15], and can adversely influence functional activities and QoL [16], decrease treatment compliance [17], and increase the risk of hospitalizations [18].

A recent systematic review reported a 45% prevalence of pain in cancer patients [3], with one-third experiencing moderate to severe pain. Another systematic review emphasizes that poorly controlled pain remains a significant challenge for many cancer patients. [19]. The context in which cancer pain is experienced and managed can vary significantly across regions, requiring localized investigations to develop effective healthcare strategies.

With a population of 30.72 million, Nepal is a low- and middle-income country (LMIC). It faces a substantial public health burden [20], with 22,008 new cancer cases and 14,704 cancer-related deaths reported in 2022, according to GLOBOCAN 2022 data [21]. The age-standardized cancer incidence and mortality rates are 81.6 and 55.3 per 100,000, respectively [21]. Lung, stomach, cervix, and uteri are the leading causes of cancer-related deaths [21]. Despite its profound impact on QoL, treatment adherence, and survival rates, the landscape of cancer pain in low-resource settings, particularly LMICs, remains less understood. Comprehensive data on cancer pain prevalence is needed to design and deliver pain services in cancer settings effectively. Challenges are due to a lack of recognition of pain as an important health challenge and its impact on QoL in LMICs.

Effective cancer pain management entails assessing pain characteristics, etiology, and specific syndromes, utilizing the Pain Management Index (PMI), a well-validated technique based on WHO guidelines [22]. The WHO recommends a 3-step pain ladder: paracetamol for mild pain, codeine for moderate pain, and morphine for severe pain [23]. Despite available guidelines, inadequate pain management persists worldwide, with reports of undertreatment globally [24, 25]. A study on the adequacy of pain management in Nepal remains lacking. Challenges to optimal cancer pain management encompass access to opioid availability [26], regulatory barriers [27], limited funding, and the absence of local pain management guidelines, alongside factors such as inadequate medical facilities [28, 29], awareness gaps [29], shortage of trained professionals [29], and limited availability of pain management close to home [30].

The primary objective of this study was to evaluate the clinical characteristics of pain and factors influencing pain intensity, as well as evaluate the association of pain severity with QoL amongst cancer patients. Secondary objectives included evaluating perceived barriers to pain management and patient medication adherence. Through these objectives, the study aims to contribute to a better understanding of cancer pain management in Nepal and enhance care delivery in oncology centers.

## Methods

### Study design and settings

The cross-sectional study was conducted at two major hospitals in Kathmandu Valley, Nepal: Kathmandu Cancer Center in Bhaktapur and Civil Service Hospital in Kathmandu. Kathmandu Cancer Center is a comprehensive private cancer center, while Civil Service Hospital is a general government hospital with an oncology wing. The data collection occurred between December 2021 and November 2022. The study adhered to the Strengthening the Reporting of Observational Studies in Epidemiology (STROBE) reporting guideline (Supplementary File 1) [31].

### Study participants and eligibility criteria

Patients were required to meet the following criteria: (1) with a documented cancer diagnosis (cancer diagnosis was confirmed through medical records and discussions with medical oncologists ensuring that all participants were aware of their condition) and be experiencing pain, (2) be at least 18 years of age and above, (3) exhibit adequate cognitive function, (4) be able to complete the questionnaires, and (5) have had no surgical procedures before the study.

### Ethical approval

Ethical approval was obtained from the Monash University Human Research Ethics Committee (MUHREC) (Project ID: 30,907) and Ethical Review Board, Nepal Health Research Council in Nepal (Ref No 768; Protocol Registration No. 497/2021). Written informed consents were obtained from all patients who met the inclusion criteria.

### Sample size

To date, there is no reported prevalence of cancer pain in Nepal. However, based on a previously reported prevalence of chronic pain of 50% in Nepal [32], the minimum required sample size for this study was calculated to estimate the prevalence of cancer pain with a 95% confidence level and a margin of error of 5%.

The sample size calculation was based on the following formula: n = [1.96^2 * p * (1 - p)] / (margin of error) ^ 2.

Where n is the sample size, 1.96 is the z-score for a 95% confidence level, p is the assumed prevalence, and the margin of error is 0.05.

Using this formula, the required sample size was calculated to be 385. This sample size was chosen to ensure sufficient power to detect the prevalence of cancer pain among the study population.

### Outcomes and outcome measures

Upon recruitment, eligible patients underwent a comprehensive paper-pencil questionnaire assessment, including socio-demographic, clinical, and psychosocial characteristics, common symptoms, QoL, pain management barriers, and medication adherence.

### Socio-demographic and clinical characteristics

Patients provided details on age, gender, education, occupation, province, annual household income, family type and marital status. The study team collected data on cancer type, stage, diagnosis, treatment history, family history, comorbidities, and Karnofsky Performance Status (KPS) [33] through patient interviews and medical records reviewed by physicians or research assistants. The severity of cancer symptoms was assessed using the validated Edmonton Symptom Assessment System – Revised (ESAS-r) [34].

### Pain characteristics

Pain intensity, location, and type were assessed. A numeric rating scale (NRS) was used to assess the pain [35]. NRS scores categorized into four pain intensity levels: no pain (score of 0 on the NRS), mild pain (scores ranging from 1 to 4), moderate pain (scores of 5 or 6), and severe pain (7–10) [36]. This assessment, administered by healthcare professionals such as clinical pharmacists, was a crucial part of the evaluation process, focusing on the patient’s pain level. Patients rated their pain intensity on the NRS, offering an immediate snapshot of their pain status.

Moreover, the classification of pain type (nociceptive or neuropathic) was determined through clinical assessments conducted by medical oncologists. Additionally, patients were asked about their experiences over the preceding 7 days to gain insight into pain characteristics, including its type. This timeframe was selected to provide a comprehensive understanding of the patient’s pain status and experiences during that period.

### Pain management index (PMI)

The PMI evaluated the adequacy of pain management based on the physician’s prescribed analgesics. Assignments were as follows: no prescribed analgesic equaled 0, a nonopioid medication (such as nonsteroidal anti-inflammatory drugs or acetaminophen) equaled 1, a weak opioid (like codeine) equaled 2, and a potent opioid (such as morphine, hydromorphone, oxycodone, or fentanyl) equaled 3 [22]. Scores were calculated by subtracting pain scores from analgesic scores, with negative scores indicating inadequate pain control.

### Quality of life (QOL) using European organization for the research and treatment of cancer quality of life questionnaire (EORTC QLQ C-30)

The validated EORTC QLQ C-30 assessed QoL in cancer patients across various domains [37]. It comprises 30 items grouped into functional and symptom scales, including physical, role, cognitive, emotional, and social functioning, as well as fatigue, pain, and nausea/vomiting symptoms. Additional items address common cancer-related symptoms and financial difficulties. Responses are on a four-point scale, except for the global health status/QoL scale, which uses a seven-point scale. Scores are transformed to a 0-100 range. Functional scales with higher scores indicate healthier functioning, while higher global health status/QoL scores signify that patients have enhanced QoL. Conversely, higher scores on symptom scales/items represent elevated symptomatology or problems. Patients completed the questionnaire and calculated scores following EORTC guidelines [37, 38]. The Nepalese version was already translated, validated, and used with permission from the EORTC [39].

### Hospital anxiety and depression scale (HADS)

The HADS evaluated anxiety and depression [40] using a 4-point Likert scale, with scores ranging from 0 to 21 for each subscale. Since the validated Nepalese version was available, its translation was incorporated into the project with permission [41].

### Barriers to pain management using barriers questionnaire (BQII)

The BQ II tool assessed barriers to optimal pain management across multiple domains: physiological effects, fatalism, communication, and harmful effects [42]. Each item is scored on a scale from 0 to 5, indicating the level of agreement with the statement, ranging from “do not agree at all” to “strongly agree” [42].

### Medication adherence using the medication adherence rating scale (MARS-5)

The MARS-5 evaluated adherence to prescribed pain medications, with scores indicating adherence levels. This validated questionnaire uses a 1-to-5 scale to assess adherence for each item (where 1 = always adherent, 5 = never adherent), yielding scores between 5 and 25. Lower scores indicate lower medication adherence. While reliable for medication adherence, the MARS-5 lacks explicit validation of cancer pain. Therefore, non-adherence was defined as a deviation from the prescribed regimen potentially affecting its intended effect [43, 44]. In this study, patients with a MARS-5 score below 20 were considered non-adherent.

### Process of data collection

Initially, medical oncologists referred patients to participate in the study. Subsequently, the research team reached out to potential participants who met the criteria recommended by the medical oncologists for data collection. During this process, the research team clearly explained the study’s objectives, emphasized participation’s voluntary and confidential nature, and obtained informed consent from willing participants.

For patients who could not read and write, trained research assistants verbally administered the questionnaires, reading out each question and recording the participant’s responses. This ensured that all participants, regardless of literacy level, could provide their input accurately and effectively. The research team also helped, and clarification as needed to ensure participants fully understood the questions.

Following consent, socio-demographic, clinical, and pain characteristic data were gathered from each participant. The study utilized several measurement instruments to assess various aspects, including the EORTC QLQ C-30, ESAS-r, BQ-II, MARS-5, and HADS.

### Addressing potential sources of bias

Several steps were undertaken to mitigate potential sources of bias in the study. A broad and diverse sample of patients from various regions of Nepal who came to Kathmandu Valley for cancer and cancer pain treatment was included to address patient selection bias. Representation from different socioeconomic backgrounds and various types and stages of cancer was ensured. To reduce information bias, outcomes were collected using standardized instruments and protocols, and all patients were assessed using the same criteria for various outcomes. For measurement bias, validated tools were used to ensure accurate measurement. To prevent reporting bias, complete and accurate reporting of all relevant data was ensured through supervision by experienced researchers.

### Data analysis

The data collected underwent rigorous analysis to inform healthcare strategies for improving cancer pain management in Nepal. Descriptive statistics were employed to analyze the frequencies, percentages, and central tendency measures for demographic, clinical, and pain characteristics. The normality of continuous variables was assessed using the Kolmogorov-Smirnov test. Descriptive statistics were also calculated for QoL scores and other relevant variables. Linear regression (stepwise) explored the associations between various predictor variables and the dependent variable, pain intensity. Initially, linear regression models were fitted to examine the unadjusted relationships between each predictor and the dependent variable. The coefficients, standard errors, t-values, p-values, and 95% confidence intervals (CI) for each predictor were obtained. Subsequently, stepwise regression was employed to identify a parsimonious set of predictors that significantly explain the variance in pain intensity. This stepwise approach allowed for automatically selecting variables based on their statistical significance, with a significance level set at 0.05. The resulting models were carefully evaluated for goodness of fit, multicollinearity, and adherence to regression assumptions. After this, linear regression was used to explore the associations between pain severity and QoL. All data were entered into the IBM Statistical Package for the Social Sciences, version 26 (SPSS, Chicago, IL).

## Results

**Fig. 1:**
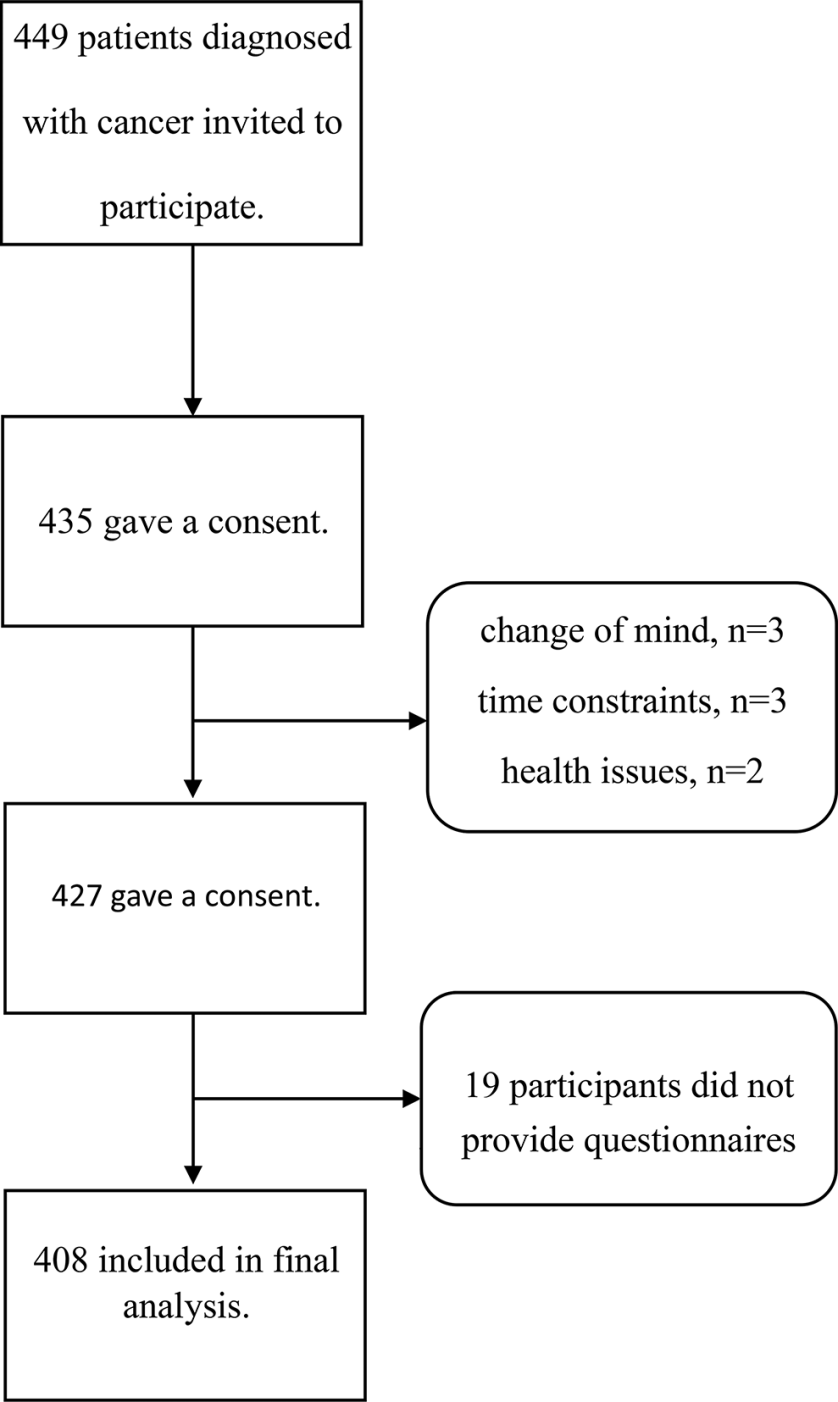
Flowchart of the study

Of the 449 individuals diagnosed with cancer, 435 (96.88%) consented to participate in the study. Eight participants withdrew before finishing the survey, citing reasons such as a “change of mind” (*n* = 3), “time constraints” (*n* = 3), and “health issues” (*n* = 2). Additionally, 19 participants did not provide responses for the study outcomes. Consequently, 408 individuals (95.5% of the initial sample) were included in the analyses (Fig. 1). Eight individuals withdrew, citing reasons such as a “change of mind” (*n* = 3), “time constraints” (*n* = 3), and “health issues” (*n* = 2). Additionally, 19 participants did not respond to the study outcomes.

### Socio-demographic data of cancer patients

Patients, with a mean age of 54.87 years, were predominantly female (55.4%) and received care at a private hospital (53.7%) and inpatient settings (70.6%) (Table 1). The education levels varied, with the majority being married (87.3%) and were from nuclear families (65.0%). As for insurance coverage, 50.0% were from the government, while 22.1% were private. Occupation-wise, 32.8% were involved in household work, 18.4% in agriculture, and 15.0% in government employment, while the rest were in other categories.

**Table 1:** Socio-demographic characteristics of patients

Characteristics	Frequency (*n*)	Percent (%)
*Age, mean ± SD, yrs. 54.87 ± 15.657*		
*Gender*		
Male	182	44.6
Female	226	55.4
*Study Site*		
Private hospital	219	53.7
Public hospital	189	46.3
*Education*		
No formal education (not able to read and write)	25	6.1
No formal education (able to read and write)	107	26.2
Primary level (1–5)	52	12.7
Lower secondary level (6–8)	27	6.6
Secondary level (8–10)	70	17.2
Intermediate level	75	18.4
Graduate	46	11.3
Postgraduate	6	1.5
*Insurance*		
Government	204	50.0
None	114	27.9
Private	90	22.1
*Province*		
Province 1 (Koshi)	47	11.5
Province 2 (Madhesh)	45	11.0
Province 3 (Bagmati)	228	55.9
Province 4 (Gandaki)	36	8.8
Province 5 (Lumbini)	33	8.1
Province 6 (Karnali)	1	0.2
Province 7 (Sudarpashchim)	18	4.4
*Household Income (per month)*		
Less than 10,000 NPR (75.16 USD)	8	2.0
10,001–50,000 NPR (75.17-375.79 USD)	123	30.1
50,001–1,00,000 NPR (375.80-751.58 USD)	119	29.2
More than 1,00,000 NPR (751.58 USD)	18	4.4
Not willing to disclose	140	34.3
*Occupation*		
Employed (Government)	61	15.0
Employed (Private)	27	6.6
Business	13	3.2
Agriculture	75	18.4
Household work	134	32.8
Wage labor	11	2.7
Unemployed	16	3.9
Student	37	9.1
Retired	34	8.3

NPR: Nepalese rupee; USD: United States Dollars

### Clinical characteristics of cancer patients

Table 2 presents a brief overview of the clinical characteristics of cancer patients with pain. Common cancer diagnoses included cervical (17.6%), lung (11.8%), and colon/rectum (12.0%) cancers. Staging distribution revealed that 7.8% were at Stage I, 35.8% at Stage II, 38.2% at Stage III, and 18.1% at Stage IV. The body mass index (BMI) classifications showed that most patients were normal weight (44.4%). Treatment modalities encompassed chemotherapy (5.9%), radiotherapy (16.4%), surgery (0.7%), and the remaining in various combinations. The purpose of treatment included curative (78.9%) and palliative (21.1%). Metastasis was observed in 18.4% of cases. KPS indicated 14.5% with scores of 10–40, 43.4% with scores of 50–70, and 42.2% with scores of 80–100. HADS showed elevated anxiety scores (> 7) in 57.6%, depression scores (> 7) in 54.9%, and a total score of > 14 in 52.5% of cases.

**Table 2:** Clinical characteristics of patients

	Frequency (*n*)	Percent (%)
*Primary Cancer Diagnosis*		
Cervical Cancer	72	17.6
Colon and rectum cancer	49	12.0
Lung Cancer	48	11.8
Brain and central nervous system cancer	41	10.0
Lip and oral cavity cancer	38	9.3
Breast Cancer	24	5.9
Larynx cancer	16	3.9
Pancreatic cancer	16	3.9
Prostate cancer	15	3.7
Oesophageal cancer	14	3.4
Stomach cancer	13	3.2
Gall Bladder Cancer	11	2.7
Thyroid Cancer	11	2.7
Non-Hodgkin lymphoma	9	2.2
Ovarian Cancer	6	1.5
Vulvar cancer	4	1.0
Nasopharynx cancer	4	1.0
Sarcoma	4	1.0
Others	4	1.0
Liver cancer	3	0.7
Uterine cancer	3	0.7
Skin Cancer	1	0.2
Unknown	1	0.2
Hodgkin Lymphoma	1	0.2
*Staging of cancer*		
I	32	7.8
II	146	35.8
III	156	38.2
IV	74	18.1
*Charlson Comorbidity Index (CCI) mean ± SD 0.52 ± 0.864*		
*Body mass index (BMI)*		
Underweight (Below 18.5)	51	12.5
Normal (18.5–24.9)	181	44.4
Overweight (25.0–29.9)	132	32.4
Obesity (30.0 and above)	44	10.8
*Treatment*		
Chemotherapy + Radiotherapy	186	45.6
Chemotherapy + Radiotherapy + Surgery	81	19.9
Radiotherapy	67	16.4
Radiotherapy + Surgery	25	6.1
Chemotherapy	24	5.9
Surgery + Chemotherapy	14	3.4
No Treatment	8	2.0
Surgery	3	0.7
*Purpose of Current Treatment*		
Curative	322	78.9
Palliative	86	21.1
*Metastasis*		
No	333	81.6
Yes	75	18.4
*Karnofsky Performance Status*		
10–40	59	14.5
50–70	177	43.4
80–100	172	42.2
*Hospital Anxiety and Depression Scale*		
HADS Anxiety score > 7	235	57.6
HADS Depression score > 7	224	54.9
HADS Total > 14	214	52.5

### Characteristics of pain in cancer patients

The pain characteristics of the patients and the analgesic classes administered are summarized in Table 3. Among the patients, the most common pain locations were in the head and neck (27.0%), followed by the abdomen (14.5%) and multiple sites (22.3%). Over half of the patients (55.6%) reported pain duration of more than 3 months. Pain duration was assessed as either greater than 3 months or less than or equal to 3 months. Regarding the type of pain, nociceptive pain was reported by 42.4% of patients, followed by neuropathic pain (26.0%) and mixed mechanisms pain (25.0%). The mean NRS for pain intensity was 4.31 (SD = 2.69), with 32.4% of patients experiencing moderate pain and 21.1% reporting severe pain.

**Table 3:** Pain characteristics of patients and analgesic’s class administered to patients in pain

	Frequency (*n*)	Percent (%)
*Pain Intensity (Current Level)*		
NRS mean (SD) of 4.31 (2.69).		
0 (No pain)	63	15.4
1–4 (Mild pain)	127	31.1
5–6 (Moderate pain)	132	32.4
7–10 (Severe pain)	86	21.1
*Pain Location* ^*a*^		
Undetermined	12	2.9
Upper limb	17	4.2
Lower limb	13	3.2
Head and neck	110	27.0
Thorax or breast	27	6.6
Back	28	6.9
Abdomen	59	14.5
Pelvis and perineum	51	12.5
Multiple sites	91	22.3
*Pain Duration* ^*b*^		
> 3 months	227	55.6
≤ 3 months	181	44.4
*Type of Pain*		
**Nociceptive**	173	42.4
Deep Somatic	76	18.6
Superficial Somatic	63	15.4
Visceral	34	8.3
**Neuropathic**	106	26.0
Peripheral Neuropathic	94	23.0
Central Neuropathic	12	2.9
Mixed	102	25.0
Undetermined	27	6.6
*Analgesic’s class administered to patients in pain*		
None	63	15.4
NSAIDs	127	31.1
Weak Opioids	132	32.4
Strong Opioids	86	21.1
*ADRs due to pain medicines*		
No ADRs	54	
ADRs Median, IQR = 2 (4,1)		
Dizziness	164	21.22
Dry mouth	132	17.08
Fatigue	109	14.10
Constipation	99	12.81
Sedation	75	9.70
Nausea	71	9.18
Vomiting	31	4.01
Delirium	22	2.85
Anxiety	17	2.20
Drowsiness	17	2.20
Insomnia	13	1.68
Itching	12	1.55
Heart burn	6	0.78
Bloating	3	0.39
Difficulty breathing	2	0.26
Total	773	

^a^ Pain location represents the primary site of pain reported by participants. Multiple locations could apply to some participants. ^b^ duration of pain experienced by the participants, not the NRS score

In this study, the distribution of the PMI among patients was as follows: 1% had a PMI of -3, 10.3% had a PMI of -2, 26% had a PMI of -1, 43.9% had a PMI of 0, 15.7% had a PMI of 1, and 3.2% had a PMI of 2 (available Supplementary Table 1). According to the definition, the number of patients with adequate pain management (PMI ≥ 0) was 56.3%, while those with inadequate pain management (PMI < 0) constituted 43.7% (Supplementary Table 1).

The majority of patients received analgesics, with NSAIDs (31.1%) and weak opioids (32.4%) being the most commonly prescribed, followed by strong opioids (21.1%). ADRs due to pain medicines were reported by a subset of patients, with dizziness (21.22%), dry mouth (17.08%), and fatigue (14.10%) being the most observed symptoms. Analgesic’s class and adjuvants administered to cancer patients experiencing pain are available in Supplementary Table 2.

### Quality of life of cancer patients

Table 4 presents the results for functional and symptom scales (single-item measures), including the mean values and the SDs. The global health status/QoL mean was 45.58 with SD of 24.16. In functional scales, CF showed a higher mean ± SD value (81.12 ± 25.47), and PF had a low mean ± SD (41.58 ± 27.52). Symptom scales included fatigue with a mean ± SD of 61.24 ± 28.093, followed by pain with a mean of 48.77. In a single item, appetite loss with a mean of 55.39, followed by financial difficulties with a mean of 55.22.

**Table 4:** Quality of life of participants using EORTC QLQ C-30

	Mean	Std. deviation
*Global health status / QoL*		
Global health status/QoL	45.58	24.16
*Functional scales*		
Physical functioning	41.58	27.52
Role functioning	40.07	30.91
Emotional functioning	62.15	25.15
Cognitive functioning	81.12	25.47
Social functioning	49.06	29.59
*Single Items*		
Fatigue	61.24	28.09
Nausea and vomiting	23.24	27.56
Pain	48.77	28.82
Dyspnoea	11.11	23.63
Insomnia	33.49	32.37
Appetite loss	55.39	36.09
Constipation	27.85	34.26
Diarrhoea	14.54	29.20
Financial difficulties	55.22	30.82

### Factors influencing the pain intensity

Patients reporting mixed mechanisms of pain exhibited significantly higher NRS scores (B = 1.458, 95% CI [0.869, 2.046], *p* < 0.001) compared to other neuropathic pain and nociceptive pain. Similarly, those experiencing pain in multiple sites reported significantly elevated NRS (B = 1.175, 95% CI [0.577, 1.773], *p* < 0.001). Lower KPS groups, specifically 10–40, were associated with higher NRS scores (B = -1.308, 95% CI [-2.010, -0.606], *p* < 0.001). Specific cancer diagnoses, including prostate cancer (B = -2.045, 95% CI [-3.338, -0.751], *p* = 0.002), pancreatic cancer (B = 1.852, 95% CI [0.592, 3.113], *p* = 0.004), oesophageal cancer (B = 1.674, 95% CI [0.363, 2.986], *p* = 0.012), and ovarian cancer (B = 1.967, 95% CI [0.022, 3.911], *p* = 0.047), were associated with varying degrees of increased NRS score. A specific treatment modality (chemotherapy + radiotherapy) was associated with a lower NRS score (B = -0.583, 95% CI [-1.062, -0.103], *p* = 0.017) compared with other treatment modalities (chemotherapy combined with radiotherapy and surgery, radiotherapy alone, radiotherapy combined with surgery, chemotherapy alone, surgery combined with chemotherapy, and surgery alone). Regional differences were observed, with residents of Province 7 (Sudarpashchim) reporting lower NRS scores, as indicated by a negative beta coefficient of -2.31 (Supplementary Table 3).

### Association of pain severity with quality of life

The findings from Table 5 demonstrated a significant association between pain severity and QoL among cancer patients. Individuals with mild, moderate, and severe pain consistently displayed diminished levels of physical functioning, role functioning, and emotional functioning compared to those without pain. Specifically, for physical functioning, individuals with mild pain exhibited a decrease of 7.64 (*p* = 0.043), while those with moderate and severe pain experienced further declines of 15.93 (*p* < 0.001) and 38.07 (*p* < 0.001) units, respectively. Similarly, increasing pain severity was associated with negative impacts on the role and emotional functioning, with coefficients ranging from − 13.73 to -39.7 and − 10.78 to -31.19, respectively (p-values < 0.05). Participants with severe pain reported significantly higher levels of fatigue (B = 26.91 *p* < 0.001), nausea and vomiting (B = 33.82, *p* < 0.001), insomnia (B = 48.33, *p* < 0.001), appetite loss (B = 39.82, *p* < 0.001), constipation (B = 27.96, *p* < 0.001), and financial difficulties (B = 7.38, *p* = 0.147) compared to those with no pain. Increasing pain severity was consistently associated with worsening symptoms across these domains (*p* < 0.05). Moreover, a significant inverse relationship was observed between pain severity and global health status/QoL (B = -37.36, *p* < 0.001), indicating that greater pain severity is linked to reduced overall well-being.

**Table 5:** Linear regression showing association of pain severity with quality of life

			Pain severity			
Functioning scale		(Constant)	None	Mild	Moderate	Severe
**Physical functioning** **(R** ^**2**^ **) = 0.217**	B	57.14	Reference	-7.64	-15.93	-38.07
	(95% CI)	(51.09, 63.2)		(-15.05, -0.23)	(-23.29, -8.57)	(-46.05, -30.1)
	Sig.	*P* < 0.001		0.043	*P* < 0.001	*P* < 0.001
**Role functioning** **(R** ^**2**^ **) = 0.166**	B (95%	60.05	Reference	-13.73	-22.68	-39.7
	CI)	(53.03, 67.07)		(-22.31, -5.14)	(-31.21, -14.15)	(-48.94, -30.47)
	Sig.	*P* < 0.001		0.002	*P* < 0.001	*P* < 0.001
**Emotional functioning** **(R** ^**2**^ **) = 0.152**	B	77.12	Reference	-10.78	-15.56	-31.19
	(95% CI)	(71.36, 82.88)		(-17.82, -3.73)	(-22.56, -8.56)	(-38.77, -23.61)
	Sig.	*P* < 0.001		0.003	*P* < 0.001	*P* < 0.001
**Cognitive functioning** **(R** ^**2**^ **) = 0.061**	B (95%	90.48	Reference	-9.77	-6.51	-19.93
	CI)	(84.34, 96.61)		(-17.27, -2.26)	(-13.97, 0.95)	(-28.01, -11.86)
	Sig.	*P* < 0.001		0.011	0.087	*P* < 0.001
**Social functioning** **(R** ^**2**^ **) = 0.085**	B	55.82	Reference	1.4	-8.22	-21.52
	(95% CI)	(48.78, 62.86)		(-7.21, 10.01)	(-16.77, 0.34)	(-30.78, -12.25)
	Sig.	*P* < 0.001		0.75	0.06	*P* < 0.001
**Symptoms Scale / Single Items**						
**Fatigue** **(R** ^**2**^ **) = 0.123**	B (95%	52.03	Reference	1.6	9.42	26.91
	CI)	(45.49, 58.57)		(-6.4, 9.6)	(1.47, 17.37)	(18.31, 35.52)
	Sig.	*P* < 0.001		0.694	0.02	*P* < 0.001
**Nausea and vomiting** **(R** ^**2**^ **) = 0.142**	B (95%	6.88	Reference	15.04	14.08	33.82
	CI)	(0.53, 13.23)		(7.27, 22.8)	(6.37, 21.8)	(25.47, 42.17)
	Sig.	0.034		*P* < 0.001	*P* < 0.001	*P* < 0.001
**Pain** **(R** ^**2**^ **) = 0.500**	B (95%	15.87	Reference	21.66	39.68	63.2
	CI)	(10.81, 20.94)		(15.46, 27.86)	(33.53, 45.84)	(56.53, 69.87)
	Sig.	*P* < 0.001		*P* < 0.001	*P* < 0.001	*P* < 0.001
**Dyspnoea** **(R** ^**2**^ **) = 0.025**	B	5.29	Reference	4.68	5.57	12.15
	(95% CI)	(-0.51, 11.09)		(-2.41, 11.78)	(-1.48, 12.62)	(4.51, 19.79)
	Sig.	0.074		0.195	0.121	0.002
**Insomnia** **(R** ^**2**^ **) = 0.235**	B	10.58	Reference	13.83	26.03	48.33
	(95% CI)	(3.54, 17.62)		(5.22, 22.44)	(17.48, 34.59)	(39.07, 57.6)
	Sig.	0.003		0.002	*P* < 0.001	*P* < 0.001
**Appetite loss** **(R** ^**2**^ **) = 0.120**	B	32.28	Reference	18.38	27.83	39.82
	(95% CI)	(23.86, 40.7)		(8.08, 28.68)	(17.59, 38.06)	(28.73, 50.9)
	Sig.	*P* < 0.001		*P* = 0.001	*P* < 0.001	*P* < 0.001
**Constipation** **(R** ^**2**^ **) = 0.063**	B	14.29	Reference	12.22	11.98	27.96
	(95% CI)	(6.04, 22.53)		(2.14, 22.31)	(1.95, 22)	(17.11, 38.82)
	Sig.	*P* = 0.001		0.018	0.019	*P* < 0.001
**Diarrhoea** **(R** ^**2**^ **) = 0.020**	B	15.34	Reference	-3.8	-3.22	6.75
	(95% CI)	(8.16, 22.53)		(-12.59, 5)	(-11.96, 5.51)	(-2.71, 16.21)
	Sig.	*P* < 0.001		0.397	0.469	0.162
**Financial difficulties** **(R** ^**2**^ **) = 0.022**	B	55.03	Reference	-5.42	1.03	7.38
	(95% CI)	(47.45, 62.6)		(-14.69, 3.85)	(-8.17, 10.24)	(-2.6, 17.35)
	Sig.	*P* < 0.001		0.251	0.825	0.147
**Global Health Status /QOL** **(R** ^**2**^ **) = 0.244**	B	64.68	Reference	-12.85	-22.32	-37.36
	(95% CI)	(59.46, 69.91)		(-19.24, -6.45)	(-28.67, -15.97)	(-44.23, -30.48)
	Sig.	*P* < 0.001		*P* < 0.001	*P* < 0.001	*P* < 0.001

### Patients’ symptom scores

**Fig. 2:**
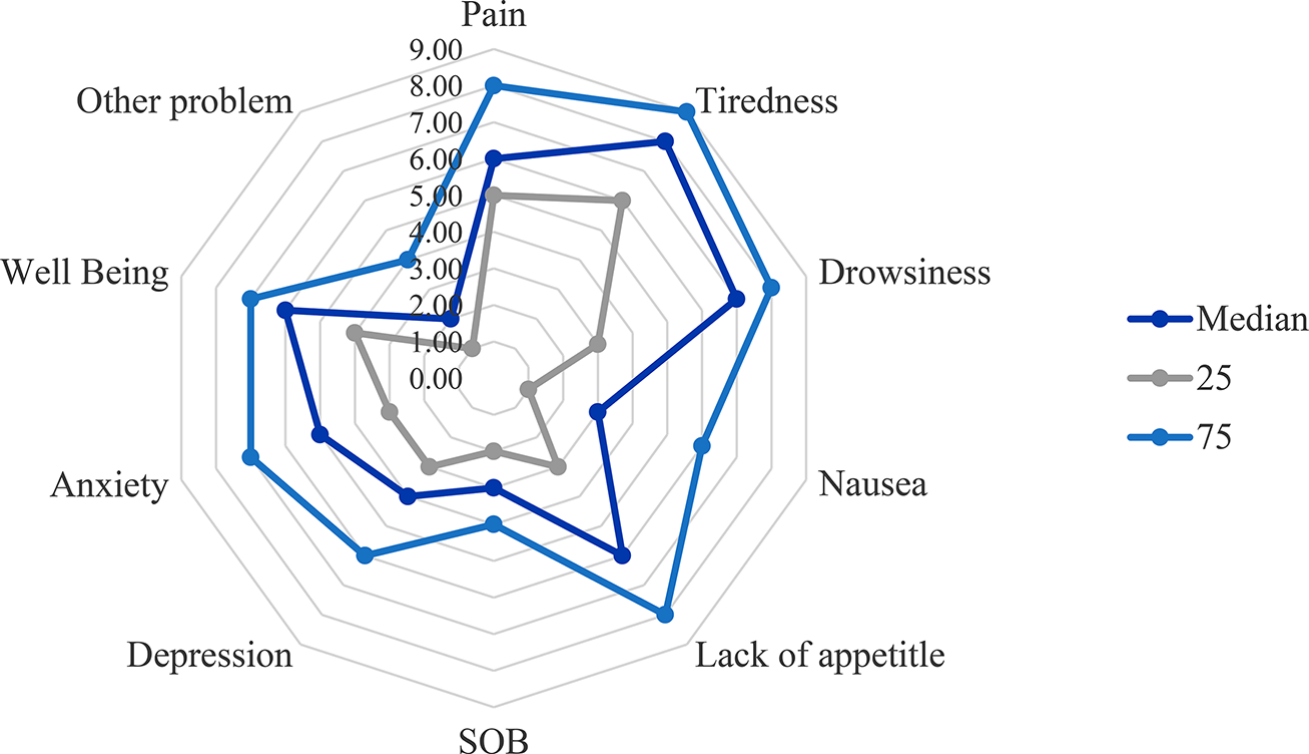
Radar chart showing ESAS-r

Patients’ symptom median scores were assessed using the Edmonton Symptom Assessment System-revised (ESAS-r) (Fig. 2). The obtained median scores indicate the symptom burden experienced by cancer treatment patients. Notably, the significant findings reveal several key symptoms with considerable severity levels, a median pain score of 6.00 (IQR: 5, 8), tiredness at 8.00 (IQR: 6, 9), and drowsiness at 7.00 (IQR: 3, 8). Notably, nausea, lack of appetite, and shortness of breath (SOB) had median scores of 3.00 (IQR: 1, 6), 6.00 (IQR: 3, 8), and 3.00 (IQR: 2, 4), respectively. Additionally, depression, anxiety, and well-being exhibited median scores of 4.00 (IQR: 3, 6), 5.00 (IQR: 3, 7), and 6.00 (IQR: 4, 7), respectively (Supplementary Table 4).

### Patients’ barriers to pain management using BQ II

Supplementary Table 5 shows patients’ pain management barriers assessed using the BQ II. The barriers are categorized into four subscales: physiological effects, fatalism, communication, and harmful effects. Within the physiological effects subscale, items such as BQ 3, BQ 5, and BQ 17 exhibited low median scores of 1.00 (IQR: 1, 4), indicating a minimal perceived influence. However, items like BQ 6, BQ 15, and BQ 22 presented moderate scores of 2.00 (IQR: 2, 4), suggesting moderate perceived barriers. In the fatalism subscale, items BQ 1, BQ 8, and BQ 24 consistently demonstrated low median scores of 0.00 (IQR: 0, 3), reflecting a generally low perception of fatalistic barriers. Communication-related barriers, as indicated by items BQ 11, BQ 18, BQ 25, BQ 26 and BQ 27, presented typically low median scores of 1.00 (IQR: 1, 4), except BQ 12, which reveals a moderate score of 3.00 (IQR: 3, 5). Finally, in the harmful effects subscale, items like BQ 2, BQ 4 and BQ 13 exhibited median scores of 2.00 (IQR: 2, 4), suggesting a moderate perception of harmful effects as barriers to pain management.

### Medication adherence among cancer patients with pain

Full adherence to the medication regimen (full score 25) was reported by 212 patients (52%). The prevalence of non-adherence (score ≥ 20) with pain medicine was 13.97%. MARS-5 score was found to vary among patients from 14 to 25. The distribution of non-adherence scores is presented in Supplementary Table 6.

## Discussion

In this study, a substantial prevalence of nociceptive pain at 42.4% and neuropathic pain at 26% among cancer patients was found, emphasizing the complexity of pain experiences within this population. Notably, this estimate of neuropathic pain prevalence falls below the lower ranges reported in existing meta-analyses and systematic reviews [19, 45]. Specifically, the prevalence of nociceptive and neuropathic pains has been documented as 59% and 40%, respectively, among cancer patients [45]. Additionally, Reis-Pina, Paulo et al. (2018) reported a prevalence of 33% for mixed neuropathic pain, characterized by both nerve and tissue damage, in cancer pain patients [46]. However, this study doesn’t establish a significant association between pain and neuropathic pain. This study found a significant association between pain and mixed mechanisms pain (nociceptive and neuropathic), which contradicted the recent findings [46–49]. Neuropathic pain, distinguished by higher pain intensity, exerts a more adverse impact on the QoL than nociceptive pain [50]. This type of pain is frequently linked to increased analgesic needs, unfavorable outcomes, and higher disability levels [50, 51]. Recognizing and distinguishing neuropathic pain from other cancer-related discomfort is crucial due to its association with more adverse pain outcomes, necessitating distinct treatment approaches [52, 53]. This finding contrasts with existing literature, highlighting the necessity for subtle pain management approaches tailored to factors such as cancer types and treatment modalities [45, 54].

The findings of this study indicated that 43.7% of patients experienced inadequate pain management, as reflected by their PMI scores. This substantial proportion highlights a critical need for improvement in pain management practices. However, it is essential to recognize that PMI alone may not fully capture the complexity of pain management, particularly in cases of short-term, non-persistent pain where morphine treatment may not be indicated. In such instances, patients may receive appropriate pain management through alternative measures, contributing to lower PMI scores without compromising the quality of care. Therefore, while PMI is a valuable indicator [22], it should be interpreted within the broader context of individual patient needs and treatment plans. Pain can be temporary, and, in such cases, high-intensity pain might not necessitate the use of strong opioids like morphine. The clinical decision to avoid strong opioids in such scenarios is appropriate to prevent unnecessary side effects and potential dependency [55]. Adequate pain management should always be guided by clinical judgment, considering the nature, duration, and intensity of the pain and the patient’s overall health status [55]. For temporary pain, short-acting opioids or non-opioid analgesics may be more appropriate, even if this results in a lower PMI score. The PMI is a useful tool for assessing the adequacy of pain control, but it has limitations [56]. Specifically, PMI may not fully reflect the adequacy of treatment for short-term pain. Patients who experience transient but severe pain may have a low PMI score if strong opioids are not prescribed despite receiving adequate treatment with other analgesics or interventions. Thus, it is important to contextualize and interpret PMI scores according to the patient’s specific pain and clinical setting. Future research should explore additional metrics and considerations to provide a more comprehensive assessment of pain management outcomes.

In countries such as Nepal with low-resource settings, it is essential to acknowledge that the findings of this study may differ from the prevalence rates reported elsewhere. The complexities of healthcare resources, accessibility, and cultural factors in low-resource settings contribute to variations in the prevalence and management of cancer-related pain. These findings underscore the importance of region-specific considerations in pain management in Nepal. Additionally, comparing the data to HICs highlights unique challenges, informing tailored interventions for better outcomes. Recognizing these differences is vital for refining global clinical practices and improving patient outcomes, particularly in low-resource settings. This study estimated an average global health status/QOL score of 45.58 among adult cancer patients experiencing pain. This is compared to another study on cancer patients with pain, reporting a mean score of 42.56, indicating a higher QOL [57]. Additionally, an average mean pain score of 48.77 was found, which indicates a moderate pain level among cancer patients in the present study. The adverse impact of severe pain on QoL is well-documented [57–62]. Although this study reported mean fatigue scores of 61.24, appetite loss scores of 55.39, and financial difficulties of 55.22, PF scores were 41.58 and RF scores of 40.0735. These findings are comparable to those reported by other studies [57, 63–67]. While patients reported moderate overall well-being, specific challenges in PF and RF and symptoms like fatigue require targeted interventions. Nevertheless, further examination is needed to untangle the multi-faceted grid of connections between cancer pain, co-occurring symptoms, and diverse QoL dimensions, warranting a multi-faceted approach.

One significant finding is the correlation between reported pain type and severity. Patients experiencing mixed pain types showed notably higher NRS scores, implying an exacerbation when neuropathic and nociceptive pain coexist. Moreover, pain location significantly impacted severity; patients with pain in multiple sites reported higher levels of NRS score compared to those with localized pain. This underscores the need to consider pain distribution in cancer pain management. Lower KPS scores, particularly 10–40, were associated with higher NRS scores, emphasizing functional impairment’s role in pain perception and the necessity for tailored interventions. Specific cancer diagnoses, such as prostate, pancreatic, oesophageal, and ovarian cancer, were linked to increased NRS scores, indicating the importance of understanding specific pain characteristics for targeted management. Interestingly, chemotherapy and radiotherapy were associated with lower NRS scores, suggesting potential pain alleviation effects warranting further investigation. Regional differences indicated that residents of Province 7 reported lower NRS scores, as evidenced by a negative beta coefficient of -2.31. However, since fewer than 5% of the sample were from this province, this result may be attributed to chance or sampling bias. Additionally, the geographical distance from Kathmandu may have resulted in a non-representative sample, potentially including wealthier individuals who could travel to the study centers. Therefore, definitive claims about geographical factors influencing pain perception are premature and should be interpreted cautiously. The ESAS-r findings symptom severity [68], with median scores for pain, tiredness, and drowsiness aligning with previous studies [69]. Notably scores were observed for nausea, lack of appetite, and shortness of breath. Depression, anxiety, and well-being also had significant impacts on patient care and symptom management. Multiple regression established significant associations between pain severity and reduced physical, role, and emotional functioning. Participants with severe pain reported significantly elevated levels of fatigue, nausea and vomiting, insomnia, appetite loss, constipation and financial difficulties. Increasing pain severity was consistently associated with worsening symptoms across these domains. These findings support the global correlation between pain and diminished QoL [61, 70–72]. The study identified varying perceptions among patients regarding barriers to pain management. Physiological effects like drowsiness, confusion, and constipation from pain medication were seen as minimally influential, while concerns about tolerance development, decreased efficacy with worsening pain, and masking of health changes were moderately perceived barriers. Fatalistic concerns were generally low, with patients expressing confidence in the relief and control of cancer pain through medication. Communication barriers were primarily low, except for moderate concern about doctors’ focus on curing illness over pain control. Moderate perceptions of harmful effects as barriers to pain management were evident, including worries about addiction, immune system weakening, and potential harm from pain medication. Comparing findings with other studies, similar communication concerns were found, but differing perceptions regarding the harmful effects of pain medicine were noted [73]. This study underscores attitudinal barriers, notably concerns about harmful effects and addiction to pain medicine, as significant obstacles to pain management [74]. Patient-related barriers, such as reluctance to report pain and misconceptions about pain medication, are identified, highlighting the importance of comprehensive patient education on cancer pain and its treatment [75]. Similar conclusions were drawn in studies conducted in Korea, Australia [76], and Jordan [77], emphasizing the harmful effects of pain medicine as significant barriers, with fears of addiction and misconceptions about pain medication being prevalent. These findings underscore the importance of addressing patient misconceptions, particularly regarding concerns about the harmful effects and addiction to pain medicine, highlighting the need for comprehensive patient education about cancer pain and its treatment.

Effective pain management in cancer relies on teamwork between healthcare professionals and patients, emphasizing the importance of medication adherence. This study examines medication non-adherence rates among cancer patients with pain, revealing varied findings across studies. While this study reports a 52% adherence rate, the adherence rates reported in other studies are remarkable. While this study reports a 52% adherence rate, others show rates ranging from 8.9% [78] to 73% [79]. Disparities in adherence rates for different analgesics highlight the need for tailored interventions. As highlighted by contrasting reports, long-term adherence considerations are crucial [80]. Addressing medication non-adherence requires tailored approaches, including patient education, addressing medication-specific concerns, and consideration of broader contextual factors to optimize pain management outcomes.

### Implications of the study

The study highlights the necessity for a longitudinal study to assess the long-term impact of pain management on the QoL of cancer patients in Nepal. It underscores the importance of tailored pain management strategies within Nepal’s healthcare context, addressing pain characteristics, adequacy of pain management, patient barriers, and medication adherence to improve overall QoL. The severity of pain significantly influences QoL, emphasizing the need for comprehensive approaches to enhance patient well-being. These findings offer valuable insights for healthcare providers and policymakers, stressing the importance of communication, education, and culturally sensitive care in optimizing pain management outcomes for cancer patients. Implementing evidence-based pain management protocols informed by these findings can significantly improve the care and well-being of cancer patients in Nepal.

### Strengths of the study

This study, the first in Nepal, has several strengths. First, a multi-center approach was utilized to draw deductions from a diverse and representative sample in the Kathmandu Valley, Nepal, and this study comprehensively explored cancer patients experiencing pain, bridging an existing research gap. Second, enrolled patients demonstrated a high compliance rate in completing the survey, and eligible patients exhibited a high participation rate. Third, this study offered a comprehensive assessment encompassing socio-demographic, clinical characteristics, pain characteristics, symptom evaluation, QoL, psychological aspect, barriers to pain management and medication adherence. Fourth, this study focuses on localized insights and emphasizes the need for more tailored healthcare strategies, acknowledging the unique challenges faced by this population. The clinical significance of this study is emphasized by evaluating the QoL, which can help provide valuable insights for optimizing the care and support offered to cancer patients experiencing pain. Fifth, the study’s strength lies in its incorporation of patients’ perspectives on barriers to pain management, offering a holistic understanding of their experiences. Sixth, using multivariable regression enhances the robustness of the findings, identifying key characteristics associated with the pain levels of cancer patients. Taken together, this study contributes valuable knowledge to improve the current understanding of cancer patients with pain, shedding light on the nature of pain and its profound implications for QoL.

### Limitations of the study

This study has several limitations. Firstly, its cross-sectional design provides a snapshot of cancer pain characteristics, but causation cannot be inferred. Secondly, the study was conducted in only two hospitals in the Kathmandu Valley, potentially limiting the representativeness of the findings for all cancer patients in Nepal. Thirdly, reliance on patient-reported outcomes raises the possibility of social desirability bias, mainly due to concerns regarding certain medications amid the global opioid epidemic. Moreover, this study excludes other treatment modalities, warranting further exploration in future research. Additionally, dichotomization of outcomes may have reduced the sensitivity and precision of the analyses. The study approach may have overlooked relevant variables in regression models, possibly leading to residual confounding. Future studies should consider additional patient-related factors (surgery timelines, timelines of pain, recall bias) and explore physician or system-related barriers to pain treatment. Despite enrolling patients from diverse care settings, the predominantly Nepalese-speaking study population may limit the generalizability of the findings. In addition to the aforementioned limitations, small sample size from Province 7 may introduce chance or sampling bias, as residents reported lower pain levels. The geographical distance from Kathmandu might have resulted in a non-representative sample, including wealthier participants. Therefore, findings on regional differences in pain perception should be interpreted cautiously. Acknowledging these limitations, the study underscores the need for targeted interventions and longitudinal assessments to understand better evolving patient perspectives on pain management barriers.

## Conclusion

In conclusion, this study highlights the multi-faceted nature of pain management and QoL for cancer patients in Nepal with low-resource settings. These findings underscore the multifactorial nature of pain perception in cancer patients, with mixed mechanisms of pain, pain in multiple sites, lower KPS, and specific cancer diagnoses, all contributing significantly to pain severity. Pain severity was linked with declining QoL. Additionally, this study emphasizes the importance of tailored interventions targeting pain characteristics, patient barriers, and medication adherence to improve overall QoL. Implementing evidence-based pain management protocols informed by these findings can significantly enhance the care and well-being of cancer patients in Nepal. Additionally, recognizing the unique cultural and socio-demographic factors that may contribute to cancer pain in Nepal requires further investigation, offering valuable insights for similar contexts in LMICs.

## Electronic supplementary material

Below is the link to the electronic supplementary material.

Supplementary Material 1
