# Supplementary material for: Comprehensive assessment of pain characteristics, quality of life, and pain management in cancer patients: a multi-center cross-sectional study
Source: Qual Life Res. 2024 Aug 6;33(10):2755–71. doi: 10.1007/s11136-024-03725-w (PMC11452497; doi:10.1007/s11136-024-03725-w)
Supplement: Supplementary file 1 — Supplementary Material 1 [file 11136_2024_3725_MOESM1_ESM.docx]

**Supplementary Table 1 Total number of patient assessments, with corresponding PMI and patient-reported pain scores**

|  | **Pain Management Index** | | | | | | **Total** |
| --- | --- | --- | --- | --- | --- | --- | --- |
|  | **-3** | **-2** | **-1** | **0** | **1** | **2** |  |
| **No pain (0)** | 0  (0%) | 0  (0%) | 0  (0%) | 47 (11.5%) | 13  (3.2%) | 3  (0.7%) | 63 (15.4%) |
| **Mild pain (1–4 )** | 0  (0%) | 0  (0%) | 30  (7.4%) | 58 (14.2%) | 29  (7.1%) | 10 (2.5%) | 127 (31.1%) |
| **Moderate pain ( 5 or 6)** | 0  (0%) | 21  (5.1%) | 52 (12.7%) | 37  (9.1%) | 22  (5.4%) | 0  (0%) | 132 (32.4%) |
| **Severe pain (7–10)** | 4  (1%) | 21  (5.1%) | 24 (5.9%) | 37  (9.1%) | 0  (0%) | 0  (0%) | 86 (21.1%) |
| **Total** | 4  (1%) | 42 (10.3%) | 106 (26%) | 179 (43.9%) | 64 (15.7%) | 13 (3.2%) | 408 (100%) |

**Supplementary Table 2 Analgesic’s class and adjuvants administered to cancer patients experiencing pain**

| **Category** | **Medicine Name** | **ATC Code** | **Quantity (n)** | **Percentage (%)** |
| --- | --- | --- | --- | --- |
| NSAIDs | Mefenamic acid | M01AG01 | 1 | 0.33 |
|  | Nimesulide | M01AX17 | 1 | 0.33 |
|  | Indomethacin | M01AB01 | 2 | 0.66 |
|  | Diclofenac | M01AB05 | 6 | 1.97 |
|  | Ibuprofen | M01AE01 | 8 | 2.62 |
|  | Aceclofenac+Paracetamol | M01AE51 | 10 | 3.28 |
|  | Ketorolac | M01AB15 | 21 | 6.89 |
|  | Etoricoxib | M01AH05 | 27 | 8.85 |
|  | Ibuprofen and paracetamol | M01AE51 | 29 | 9.51 |
|  | Acelofenac | M01AB15 | 31 | 10.16 |
|  | Paracetamol | N02BE01 | 123 | 40.33 |
| Weak Opioids | Codeine | N02AA59 | 12 | 9.6 |
|  | Tapentadol | N02AX06 | 12 | 9.6 |
|  | Tramadol | N02AX02 | 29 | 23.2 |
|  | Tramadol + Acetaminophen | N02AJ13 | 72 | 57.6 |
| Strong Opioids | Fentanyl | N02AB03 | 10 | 12.05 |
|  | Pethidine | N02AB02 | 4 | 4.82 |
|  | Morphine | N02AA01 | 69 | 83.13 |
| Adjuvants | Cinnarizine | N07CA02 | 7 | 3 |
|  | Cortisone | H02AB10 | 1 | 0.43 |
|  | Glucocorticoid | H02AB06 | 1 | 0.43 |
|  | Methyl folate + Methylcobalamin | B03BB03 | 1 | 0.43 |
|  | Methylcobalamin + Pregabalin | N03AX16 | 5 | 2.15 |
|  | Amitriptyline | N06AA09 | 7 | 3 |
|  | Pregabalin | N03AX16 | 5 | 2.15 |
|  | Clonazepam | N03AE01 | 6 | 2.58 |
|  | Haloperidol | N05AD01 | 8 | 3.43 |
|  | Baclofen | M03BX01 | 3 | 1.29 |
|  | Gabapentin | N03AX12 | 27 | 11.58 |
|  | Drotaverine | A03AD02 | 4 | 1.72 |
|  | Bisphosphonates | M05BA | 3 | 1.29 |
|  | Diazepam | N05BA01 | 10 | 4.29 |
|  | Prednisolone | H02AB06 | 11 | 4.72 |
|  | Hyoscine butylbromide | A03BB01 | 12 | 5.15 |
|  | Leveracetam | N03AX14 | 14 | 6.01 |
|  | Hydrocortisone | H02AB09 | 17 | 7.3 |
|  | Methylcobalamin | B03BA05 | 20 | 8.58 |

NSAIDs Non-steroidal anti-inflammatory drugs; ATC Code Anatomical Therapeutic Chemical Code

**Supplementary Table 3 Linear regression showing factors predicting the pain intensity of patients**

| **Model** |  | **Unstandardized Coefficients (B)** | **Std. Error** | **95 % CI** | **Sig.** |
| --- | --- | --- | --- | --- | --- |
| 1 | (Constant) | 3.94 | 0.15 | 3.65, 4.24 | p < 0.001 |
|  | Pain type: Mixed | 1.46 | 0.3 | 0.87, 2.05 | p < 0.001 |
| 2 | (Constant) | 3.7 | 0.16 | 3.39, 4.01 | p < 0.001 |
|  | Pain type: Mixed | 1.31 | 0.3 | 0.72, 1.89 | p < 0.001 |
|  | Location of pain: Multiple sites | 1.26 | 0.31 | 0.65, 1.86 | p < 0.001 |
| 3 | (Constant) | 3.9 | 0.17 | 3.58, 4.23 | p < 0.001 |
|  | Pain type: Mixed | 1.34 | 0.29 | 0.76, 1.91 | p < 0.001 |
|  | Location of pain: Multiple sites | 1.18 | 0.3 | 0.58, 1.77 | p < 0.001 |
|  | KPS: 10-40 | -1.31 | 0.36 | -2.01, -0.61 | p < 0.001 |
| 4 | (Constant) | 4.38 | 0.22 | 3.94, 4.81 | p < 0.001 |
|  | Pain type: Mixed | 1.21 | 0.29 | 0.64, 1.79 | p < 0.001 |
|  | Location of pain: Multiple sites | 1.11 | 0.3 | 0.52, 1.7 | p < 0.001 |
|  | KPS: 10-40 | -1.74 | 0.38 | -2.48, -1 | p < 0.001 |
|  | KPS: 80-100 | -0.87 | 0.27 | -1.41, -0.34 | p = 0.001 |
| 5 | (Constant) | 4.49 | 0.22 | 4.06, 4.93 | p < 0.001 |
|  | Pain type: Mixed | 1.22 | 0.29 | 0.66, 1.79 | p < 0.001 |
|  | Location of pain: Multiple sites | 1.01 | 0.3 | 0.42, 1.6 | p = 0.001 |
|  | KPS: 10-40 | -1.71 | 0.37 | -2.44, -0.97 | p < 0.001 |
|  | KPS: 80-100 | -0.94 | 0.27 | -1.47, -0.41 | p = 0.001 |
|  | Cancer diagnosis: Prostrate | -2.04 | 0.66 | -3.34, -0.75 | 0.002 |
| 6 | (Constant) | 4.38 | 0.22 | 3.94, 4.82 | p < 0.001 |
|  | Pain type: Mixed | 1.34 | 0.29 | 0.77, 1.91 | p < 0.001 |
|  | Location of pain: Multiple sites | 0.96 | 0.3 | 0.38, 1.55 | p = 0.001 |
|  | KPS: 10-40 | -1.63 | 0.37 | -2.37, -0.9 | 0 |
|  | KPS: 80-100 | -0.93 | 0.27 | -1.45, -0.4 | p = 0.001 |
|  | Cancer diagnosis: Prostrate | -2.07 | 0.65 | -3.35, -0.78 | 0.002 |
|  | Type of pain : Undetermined | 1.29 | 0.5 | 0.31, 2.27 | p = 0.001 |
| 7 | (Constant) | 4.41 | 0.22 | 3.97, 4.85 | p < 0.001 |
|  | Pain type: Mixed | 1.34 | 0.29 | 0.78, 1.9 | p < 0.001 |
|  | Location of pain: Multiple sites | 0.9 | 0.3 | 0.31, 1.48 | 0.003 |
|  | KPS: 10-40 | -1.51 | 0.37 | -2.24, -0.78 | p < 0.001 |
|  | KPS: 80-100 | -0.85 | 0.27 | -1.37, -0.32 | 0.002 |
|  | Cancer diagnosis: Prostrate | -2.16 | 0.65 | -3.43, -0.88 | p = 0.001 |
|  | Type of pain : Undetermined | 1.43 | 0.5 | 0.45, 2.4 | 0.004 |
|  | Province: Province 7 (Sudarpashchim) | -1.71 | 0.6 | -2.88, -0.53 | 0.005 |
| 8 | (Constant) | 4.35 | 0.22 | 3.91, 4.78 | p < 0.001 |
|  | Pain type: Mixed | 1.24 | 0.29 | 0.68, 1.8 | p < 0.001 |
|  | Location of pain: Multiple sites | 0.92 | 0.29 | 0.34, 1.5 | 0.002 |
|  | KPS: 10-40 | -1.55 | 0.37 | -2.28, -0.83 | p < 0.001 |
|  | KPS: 80-100 | -0.76 | 0.27 | -1.29, -0.24 | 0.004 |
|  | Cancer diagnosis: Prostrate | -2.07 | 0.64 | -3.33, -0.8 | p = 0.001 |
|  | Type of pain: Undetermined | 1.35 | 0.49 | 0.38, 2.31 | 0.006 |
|  | Province: Province 7 (Sudarpashchim) | -2.06 | 0.61 | -3.26, -0.87 | p = 0.001 |
|  | Cancer Diagnosis: Pancreatic | 1.85 | 0.64 | 0.59, 3.11 | 0.004 |
| 9 | (Constant) | 4.35 | 0.22 | 3.92, 4.78 | p < 0.001 |
|  | Pain type: Mixed | 1.19 | 0.29 | 0.63, 1.75 | p < 0.001 |
|  | Location of pain: Multiple sites | 0.83 | 0.29 | 0.25, 1.4 | 0.005 |
|  | KPS: 10-40 | -1.68 | 0.37 | -2.41, -0.96 | p < 0.001 |
|  | KPS: 80-100 | -0.81 | 0.26 | -1.33, -0.29 | 0.002 |
|  | Cancer diagnosis: Prostrate | -2.02 | 0.64 | -3.27, -0.76 | 0.002 |
|  | Type of pain: Undetermined | 1.38 | 0.49 | 0.42, 2.35 | 0.005 |
|  | Province: Province 7 (Sudarpashchim) | -2.01 | 0.6 | -3.2, -0.83 | p = 0.001 |
|  | Cancer Diagnosis: Pancreatic | 1.92 | 0.64 | 0.67, 3.17 | 0.003 |
|  | Cancer Diagnosis: Oesophageal | 1.67 | 0.67 | 0.36, 2.99 | 0.012 |
| 10 | (Constant) | 4.61 | 0.25 | 4.13, 5.1 | p < 0.001 |
|  | Pain type: Mixed | 1.21 | 0.28 | 0.66, 1.77 | p < 0.001 |
|  | Location of pain: Multiple sites | 0.84 | 0.29 | 0.27, 1.42 | 0.004 |
|  | KPS: 10-40 | -1.74 | 0.37 | -2.46, -1.01 | p < 0.001 |
|  | KPS: 80-100 | -0.79 | 0.26 | -1.31, -0.28 | 0.003 |
|  | Cancer diagnosis: Prostrate | -2.12 | 0.64 | -3.38, -0.87 | p = 0.001 |
|  | Type of pain: Undetermined | 1.46 | 0.49 | 0.51, 2.42 | 0.003 |
|  | Province: Province 7 (Sudarpashchim) | -2.15 | 0.6 | -3.34, -0.97 | p < 0.001 |
|  | Cancer Diagnosis: Pancreatic | 1.76 | 0.64 | 0.5, 3.01 | 0.006 |
|  | Cancer Diagnosis: Oesophageal | 1.82 | 0.67 | 0.51, 3.13 | 0.007 |
|  | Treatment: Chemotherapy + Radiotherapy | -0.58 | 0.24 | -1.06, -0.1 | 0.017 |
| 11 | (Constant) | 4.62 | 0.24 | 4.14, 5.1 | p < 0.001 |
|  | Pain type: Mixed | 1.27 | 0.28 | 0.71, 1.82 | p < 0.001 |
|  | Location of pain: Multiple sites | 0.81 | 0.29 | 0.24, 1.39 | 0.005 |
|  | KPS: 10-40 | -1.77 | 0.37 | -2.49, -1.04 | p < 0.001 |
|  | KPS: 80-100 | -0.82 | 0.26 | -1.33, -0.31 | 0.002 |
|  | Cancer diagnosis: Prostrate | -2.14 | 0.63 | -3.39, -0.9 | p = 0.001 |
|  | Type of pain: Undetermined | 1.46 | 0.49 | 0.51, 2.42 | 0.003 |
|  | Province: Province 7 (Sudarpashchim) | -2.15 | 0.6 | -3.32, -0.97 | p < 0.001 |
|  | Cancer Diagnosis: Pancreatic | 1.73 | 0.63 | 0.48, 2.98 | 0.007 |
|  | Cancer Diagnosis: Oesophageal | 1.8 | 0.66 | 0.5, 3.11 | 0.007 |
|  | Treatment: Chemotherapy + Radiotherapy | -0.55 | 0.24 | -1.03, -0.08 | 0.023 |
|  | Cancer Diagnosis: Skin | -5.34 | 2.39 | -10.04, -0.63 | 0.026 |
| 12 | (Constant) | 4.45 | 0.26 | 3.95, 4.96 | p < 0.001 |
|  | Pain type: Mixed | 1.22 | 0.28 | 0.66, 1.77 | p < 0.001 |
|  | Location of pain: Multiple sites | 0.82 | 0.29 | 0.25, 1.39 | 0.005 |
|  | KPS: 10-40 | -1.89 | 0.37 | -2.62, -1.16 | p < 0.001 |
|  | KPS: 80-100 | -0.88 | 0.26 | -1.39, -0.36 | p = 0.001 |
|  | Cancer diagnosis: Prostrate | -2.13 | 0.63 | -3.37, -0.89 | 0.001 |
|  | Type of pain: Undetermined | 1.35 | 0.49 | 0.4, 2.31 | 0.006 |
|  | Province: Province 7 (Sudarpashchim) | -2.19 | 0.6 | -3.36, -1.01 | p < 0.001 |
|  | Cancer Diagnosis: Pancreatic | 1.75 | 0.63 | 0.5, 2.99 | 0.006 |
|  | Cancer Diagnosis: Oesophageal | 1.8 | 0.66 | 0.51, 3.1 | 0.007 |
|  | Treatment: Chemotherapy + Radiotherapy | -0.6 | 0.24 | -1.07, -0.12 | 0.015 |
|  | Cancer Diagnosis: Skin | -5.58 | 2.39 | -10.27, -0.89 | 0.02 |
|  | Insurance: Government insurance | 0.51 | 0.24 | 0.04, 0.98 | 0.035 |
| 13 | (Constant) | 4.41 | 0.26 | 3.9, 4.91 | p < 0.001 |
|  | Pain type: Mixed | 1.25 | 0.28 | 0.69, 1.8 | p < 0.001 |
|  | Location of pain: Multiple sites | 0.74 | 0.29 | 0.16, 1.31 | 0.012 |
|  | KPS: 10-40 | -1.83 | 0.37 | -2.56, -1.11 | p < 0.001 |
|  | KPS: 80-100 | -0.82 | 0.26 | -1.34, -0.31 | 0.002 |
|  | Cancer diagnosis: Prostrate | -2.12 | 0.63 | -3.36, -0.88 | p = 0.001 |
|  | Type of pain: Undetermined | 1.41 | 0.48 | 0.46, 2.37 | 0.004 |
|  | Province: Province 7 (Sudarpashchim) | -2.31 | 0.6 | -3.49, -1.14 | p < 0.001 |
|  | Cancer Diagnosis: Pancreatic | 1.8 | 0.63 | 0.56, 3.03 | 0.005 |
|  | Cancer Diagnosis: Oesophageal | 1.84 | 0.66 | 0.55, 3.13 | 0.005 |
|  | Treatment: Chemotherapy + Radiotherapy | -0.6 | 0.24 | -1.08, -0.13 | 0.013 |
|  | Cancer Diagnosis: Skin | -5.55 | 2.38 | -10.22, -0.88 | 0.02 |
|  | Insurance: Government insurance | 0.5 | 0.24 | 0.03, 0.97 | 0.038 |
|  | Cancer diagnosis: Ovarian | 1.97 | 0.99 | 0.02, 3.91 | 0.047 |

KPS Karnofsky Performance Status

**Supplementary Table 4 Individual symptom median scores of patients using ESAS-r**

| **ESAS-r items** | **Median** | **IQR** | **Bootstrap^a^** | **95% Confidence Interval** | |
| --- | --- | --- | --- | --- | --- |
|  |  |  | **Bias** | **Lower** | **Upper** |
| Pain | 6.00 | (5, 8) | 0.00 | 6.00 | 6.00 |
| Tiredness | 8.00 | (6, 9) | -0.03 | 7.50 | 8.00 |
| Drowsiness | 7.00 | (3, 8) | -0.22 | 6.00 | 7.00 |
| Nausea | 3.00 | (1, 6) | -0.26 | 2.00 | 3.00 |
| Lack of appetite | 6.00 | (3, 8) | -0.17 | 5.00 | 7.00 |
| SOB | 3.00 | (2, 4) | -0.01 | 3.00 | 3.00 |
| Depression | 4.00 | (3, 6) | 0.00 | 4.00 | 4.00 |
| Anxiety | 5.00 | (3, 7) | -0.14 | 4.00 | 5.00 |
| Well-Being | 6.00 | (4, 7) | -0.43 | 5.00 | 6.00 |
| Other problem | 2.00 | (1, 4) | 0.19 | 2.00 | 3.00 |

ESAS-r Edmonton Symptom Assessment System Revised; ^a^ Unless otherwise noted, bootstrap results are

based on 1000 bootstrap samples; IQR Interquartile range

**Supplementary Table 5 Patient Barriers of Pain Management Using BQ II**

| **Subscale** | **Items** | **Median** | **IQR** | **Bootstrap^a^** | **95% Confidence Interval** | |
| --- | --- | --- | --- | --- | --- | --- |
|  |  |  |  | **Bias** | **Lower** | **Upper** |
| Physiological Effects | BQ 3  Drowsiness from pain medicine is difficult to control. | 1.00 | (1, 4) | 0.00 | 3.00 | 3.00 |
|  | BQ 5  Confusion from pain medicine can not be controlled. | 1.00 | (1, 4) | 0.00 | 3.00 | 3.00 |
|  | BQ 6  When you use pain medicine your body becomes used to its effects and pretty soon it won't work any more | 2.00 | (2, 4) | 0.00 | 3.00 | 3.00 |
|  | BQ 7  Using pain medicine blocks your ability to know if you have any new pain. | 2.00 | (2, 4) | 0.06 | 3.00 | 4.00 |
|  | BQ 10  Nausea from pain medicine can not be relieved. | 1.00 | (1, 3) | -0.05 | 1.00 | 2.00 |
|  | BQ 14  Pain medicine makes you say or do embarrassing things. | 1.00 | (1, 3) | -0.31 | 1.00 | 2.00 |
|  | BQ 15  If you take pain medicine when you have some pain, then it might not work as well if the pain becomes worse | 2.00 | (2, 4) | 0.00 | 3.00 | 3.00 |
|  | BQ 16  Pain medicine can keep you from knowing what's going on in your body | 2.00 | (2, 4) | 0.00 | 3.00 | 3.00 |
|  | BQ 17  Constipation from pain medicine can not be relieved. | 1.00 | (1, 3) | 0.15 | 1.00 | 2.00 |
|  | BQ 20  It is easier to put up with pain than with the side effects that come from pain medicine | 0.00 | (0, 3) | 0.05 | 1.00 | 2.00 |
|  | BQ 21  If you use pain medicine now, it won't work as well if you need it later | 2.00 | (2, 4) | 0.02 | 3.00 | 3.00 |
|  | BQ 22  Pain medicine can mask changes in your health | 2.00 | (2, 4) | 0.00 | 3.00 | 3.00 |
| Fatalism | BQ 1  Cancer pain can be relieved. | 0.00 | (0, 3) | 0.20 | 1.00 | 2.00 |
|  | BQ 8  Pain medicine can effectively control cancer pain | 1.00 | (1, 3) | 0.00 | 2.00 | 2.00 |
|  | BQ 24  Medicine can relieve cancer pain | 1.00 | (1, 3) | 0.15 | 1.00 | 2.00 |
| Communication | BQ 11  It is important to be strong by not talking about pain | 1.00 | (1, 4) | -0.22 | 2.00 | 3.00 |
|  | BQ 12  It is important for the doctor to focus on curing illness, and not waste time  controlling pain | 3.00 | (3, 5) | 0.00 | 4.00 | 4.00 |
|  | BQ 18  If doctors have to deal with pain they won't concentrate on curing the disease | 1.00 | (1, 4) | 0.00 | 3.00 | 3.00 |
|  | BQ 25  Doctors might find it annoying to be told about pain | 1.00 | (1, 3) | -0.01 | 2.00 | 2.00 |
|  | BQ 26  Reports of pain could distract a doctor from curing the cancer. | 1.00 | (1, 4) | 0.01 | 2.00 | 2.00 |
|  | BQ 27  If I talk about pain, people will think I'm a complainer. | 1.00 | (1, 4) | 0.28 | 2.00 | 3.00 |
| Harmful Effects | BQ 2  There is a danger of becoming addicted to pain medicine | 2.00 | (2, 4) | 0.00 | 3.00 | 3.00 |
|  | BQ 4  Pain medicine weakens the immune system | 2.00 | (2, 4) | 0.02 | 3.00 | 3.00 |
|  | BQ 9  Many people with cancer get addicted to pain medicine | 1.00 | (1, 4) | 0.00 | 3.00 | 3.00 |
|  | BQ 13  Using pain medicine can harm your immune system | 2.00 | (2, 4) | 0.00 | 3.00 | 3.00 |
|  | BQ 19  Pain medicine can hurt your immune system | 2.00 | (2, 4) | 0.00 | 3.00 | 3.00 |
|  | BQ 23  Pain medicine is very addictive | 1.25 | (1.25, 4) | 0.00 | 3.00 | 3.00 |

^a^ Unless otherwise noted, bootstrap results are based on 1000 bootstrap samples; IQR Interquartile range

**Supplementary Table 6. Distribution and frequency of MARS scores**

| **MARS-5 score** | **Frequencies (n)** | **%** |
| --- | --- | --- |
| 14 | 14 | 3.4 |
| 15 | 41 | 10.0 |
| 19 | 1 | 0.2 |
| 20 | 1 | 0.2 |
| 21 | 73 | 17.9 |
| 22 | 22 | 5.4 |
| 23 | 28 | 6.9 |
| 24 | 16 | 3.9 |
| 25 | 212 | 52.0 |
